# “Lives in the balance”: The politics of integration in the Partnership for Maternal, Newborn and Child Health

**DOI:** 10.1093/heapol/czw023

**Published:** 2016-04-22

**Authors:** Katerini T Storeng, Dominique P Béhague

**Affiliations:** ^1^Centre for Development and the Environment, University of Oslo, Oslo, Norway; ^2^London School of Hygiene & Tropical Medicine, London, UK; ^3^The Center for Medicine, Health and Society, Vanderbilt University, USA; ^4^Department of Social Science, Health and Medicine, Kings College London, London, UK

**Keywords:** Advocacy coalitions, global health, integration, maternal health, public-private partnerships, safe motherhood

## Abstract

A decade ago, the Partnership for Maternal, Newborn and Child Health (PMNCH) was established to combat the growing fragmentation of global health action into uncoordinated, issue-specific efforts. Inspired by dominant global public-private partnerships for health, the PMNCH brought together previously competing advocacy coalitions for safe motherhood and child survival and attracted support from major donors, foundations and professional bodies. Today, its founders highlight its achievements in generating priority for ‘MNCH’, encouraging integrated health systems thinking and demonstrating the value of collaboration in global health endeavours. Against this dominant discourse on the success of the PMNCH, this article shows that rhetoric in support of partnership and integration often masks continued structural drivers and political dynamics that bias the global health field towards vertical goals. Drawing on ethnographic research, this article examines the Safe Motherhood Initiative’s evolution into the PMNCH as a response to the competitive forces shaping the current global health field. Despite many successes, the PMNCH has struggled to resolve historically entrenched programmatic and ideological divisions between the maternal and child health advocacy coalitions. For the Safe Motherhood Initiative, the cost of operating within an extremely competitive policy arena has involved a partial renouncement of ambitions to broader social transformations in favour of narrower, but feasible and ‘sellable’ interventions. A widespread perception that maternal health remains subordinated to child health even within the Partnership has elicited self-protective responses from the safe motherhood contingent. Ironically, however, such responses may accentuate the kind of fragmentation to global health governance, financing and policy solutions that the Partnership was intended to challenge. The article contributes to the emerging critical ethnographic literature on global health initiatives by highlighting how integration may only be possible with a more radical conceptualization of global health governance.

Key Messages

- The Partnership for Maternal, Newborn and Child Health (PMNCH) was formed to combat fragmentation in global health.
- Despite many successes, the PMNCH’s attempt to foster partnerships and advance an integrated global policy approach generated competitive tensions and elicited self-protective responses.
- Rhetoric in support of integration often masks continued structural drivers and political dynamics that bias the global health field towards vertical goals.

## Introduction

In 1987, the Safe Motherhood Initiative (SMI) was launched to bring attention to the ‘neglected tragedy’ of maternal mortality, amidst frustration that the M in MCH—maternal and child health—had been marginalized by the prevailing focus on child survival in international health efforts (Rosenfield and Maine 1985; Starrs 1987). Ironically, in 2005, the Partnership for Maternal, Newborn and Child Health (PMNCH) was formed at the ‘Lives in the Balance’ meeting in New Delhi to bring maternal and child initiatives back together. Its mission was ‘to support the global health community to work successfully towards achieving MDG 4 and 5’ on child survival and maternal health (PMNCH 2009a, p. 12). In the decade since, the PMNCH has grown from 80 to over 500 members, attracted donor funding from governments, multilateral organizations and private foundations, and spawned a number of high-profile global initiatives (PMNCH 2014).

The rise of the PMNCH is but one example of a much larger trend driven by two potentially contradictory forces. First is the long-standing recognition that global health initiatives have often been unproductively fragmented according to disease-based expertise and that to remedy this problem, greater attention to ‘integration’ at the level of policy, governance, financing strategies, research and actual programme implementation is needed (Travis *et al.* 2004; McCoy 2009; Atun *et al.* 2010). Much of this debate about integration concerns how to reconcile the tension between narrowly targeted interventions and those providing broader system-wide support (Buffardi 2014). The second force is the rapid proliferation during the past 15 years of global public-private partnerships (PPPs) that invest in specific diseases and interventions through joint decision-making among multiple partners from the public and private sectors, including multilateral agencies, donor bodies, philanthropic foundations and civil society (Reich 2002; Buse and Harmer 2007; Harmer 2011). While most focus on single diseases, some PPPs—like the PMNCH—have explicitly organized their work to reduce duplication of efforts by creating initiatives that bring together different groups of disease-specialists to better coordinate efforts under the same programmatic umbrella. This networking of resources and expertise—what political scientists call the production of ‘transnational advocacy coalitions’ (Sabatier and Jenkins-Smith 1993; Stone 2002)—has been highly successful both politically and financially (Shiffman *et al.* 2015). The two largest partnerships, the Global Fund and GAVI (the Vaccine Alliance), almost single-handedly brought about the 4-fold increase in development assistance to the health sector between 1999 and 2005 (Ravishankar *et al.* 2009).

Despite such successes at a global level, critics have been quick to demonstrate that the business-oriented ethos that PPPs typically endorse has in fact amplified competition between initiatives, increased bureaucratization and further fragmented global health governance on the ground, thereby undermining coordinated and sustainable health action (Béhague *et al.* 2009; McCoy 2009; Taylor and Harper 2014). This fragmentation, many argue, results from the increased agenda-setting power of private foundations, notably the Bill and Melinda Gates Foundation, which has undermined the ability of publicly mandated institutions like the World Health Organization and recipient governments to promote integrated health policies, threatening democratic global health governance (Cueto 2004; Brown *et al.* 2006; McCoy and McGoey 2011; Birn 2014). Moreover, ethnographic studies have demonstrated that private foundations’ success in shaping health policy in line with their convictions has often been achieved by limiting their focus to ‘safe issues’ and marginalizing other perspectives, as in the case of malaria (Eckl 2014), or by appropriating broader policy agendas, as in the case of GAVI’s health system strengthening investment (Storeng 2014). An emerging body of social scientific research points to the detrimental effects of friction among ‘partners’ at global, national and sub-national levels, for instance the way in which civil society organizations’ weak position within PPPs limits their impact on national policy-making, and can result in co-option of progressive discourses by stronger partners and stifling of critical voices (Doyle and Patel 2008; Harmer *et al.* 2012; Kapilashrami and McPake 2012; Kapilashrami and O'Brien 2012; Grebe 2015). Today, key donors and governments embrace the rhetoric of ‘integration’ at multiple levels, but they continue to favour issue-specific partnerships and judge success in terms of disease-specific performance (Birn 2009), without acknowledging the potential contradiction at play.

On the spectrum of different kinds of PPPs, the PMNCH has in many ways been at the forefront of explicitly addressing the contradictions that privatization of global health poses for coordinated global health governance, for its *raison d’etre* was ostensibly to resolve tensions that had been dividing the maternal and child health communities since the 1980s. Flavia Bustreo, the Partnership’s director from 2006 to 2010, has argued that ‘the potential of the PMNCH to unify the previously fragmented maternal, newborn and child health (MNCH) communities created a positive atmosphere and sparked productive collaborations across sectors and constituencies’ (Bustreo *et al.* 2012, p. S7). The increased visibility of the ‘MNCH’ agenda, she argues, helped promote a ‘continuum of care’ approach in maternal, newborn and child health care initiatives, thereby fostering greater appreciation for integrated health systems thinking (Bustreo *et al.* 2012, p. S7).

Against this dominant narrative on the PMNCH’s success, this article examines ethnographically the practical tensions and contestations that have emerged as key PMNCH actors attempt to put the ideals of ‘partnership’ and ‘integration’ into practice. While recognizing that integration of services on the ground is extremely complex (Atun *et al.* 2010; Church *et al.* 2015), we seek to understand how the partnership ideal works out in terms of integration of institutional structures and policy approaches at the global level. Did the PMNCH in the end help integrate divisive groups of experts in the global arena? Did it advance an integrated policy approach? Through an ethnographic case study of the SMI’s growth and evolution into the PMNCH, we aim to show that the PMNCH has struggled to resolve historically entrenched differences in public health ideology and approaches between the maternal and child health communities. These relate to the relative emphasis on comprehensive versus selective approaches to health and the role of long-term health system development versus the pursuit of magic bullets. Despite recent claims that the PMNCH is ‘a testament to growing emphasis on collaboration as the most effective way forward to improving the lives of women, newborn and children’ (Bustreo *et al.* 2012, p. 8), we explore how the politics of institutional control and distinct advocacy coalitions’ tendency towards competition for funding and attention have posed a challenge to effective collaboration—or partnership—between expert groups. We end by reflecting on whether the current partnership model that dominates global health governance is equipped to meet the challenges of achieving institutional and programmatic integration.

## Methods

This article draws on a broader historical ethnography of the SMI (Béhague and Storeng 2008; Storeng 2010), carried out between 2004 and 2009, when the SMI was evolving into the PMNCH. Our analysis draws on participant observation within research communities and at policy events and in-depth confidential interviews with 72 informants from multilateral agencies, academic institutions, professional bodies and international non-governmental organizations, primarily at the global level (see Table 1). Most considered themselves maternal health experts, but some had expertise in child health, health systems and policy. Many of our informants were directly involved in the PMNCH as secretariat staff, donors or committee representatives. They provided an insider perspective, while data from participant observation and interviews with other global health experts provided an outsider perspective. The interviews were open-ended, and covered professional trajectories, perspectives and experiences of the historic tensions between the maternal and child health fields, the SMI’s history and identity, the formation and success of the PMNCH, responses to the evidence-based policy-making movement and the impact of competition on research and policy. During our fieldwork, we also worked as researchers within an interdisciplinary Research Programme Consortium on maternal and newborn health funded by the UK Department for International Development (DFID), which gave us access to debates about the PMNCH as they played out within expert communities and at focusing events, such as the first PMNCH high-level meeting in India in 2005. To examine the SMI’s development into the PMNCH, we also draw on historical and contemporary policy documents and scientific literature and oral histories collected during in-depth interviews. For further details of the methods please see Béhague and Storeng 2008 and Storeng and Béhague 2014.

**Table 1.** Distribution of informants according to role at the time of the interview

Role	Number
United Nations agency officials	12
Bilateral agency officials	11
International academic researchers	23
NGOs or foundation representatives	17
National-level policy makers, programme managers and researchers	9
Total	72

## Results

### The SMI’s contested beginnings

The emergence of the PMNCH can best be understood within the contested beginnings of the SMI, and particularly the ideological and programmatic differences that polarized the maternal and child health communities during the 1980s. Healthcare for mothers and children—so-called MCH services—had been at the heart of the 1978 Alma-Ata Declaration on Primary Health Care (PHC) in 1978 (WHO and UNICEF 1978). However, according to informants who had worked within international health at the time, by the early 1980s international public health specialists started voicing concern that maternal health was being neglected in favour of child health. This neglect was part of a broader backlash against the principles of the Alma-Ata declaration initiated by governments, agencies and individuals who proposed ‘selective’ PHC as a more pragmatic, financially palatable, and politically unthreatening alternative to comprehensive PHC (Cueto 2004). Critics have argued that selective PHC, being focused on a small number of cost-effective interventions only, reduced Alma-Ata’s idealism to ‘a practical set of technical interventions whose implementation and effects could be readily measured’ (Brown *et al.* 2006, p. 67) and abandoned its focus on equity and health systems development (Magnussen *et al.* 2004), aspects that maternal health specialists were particularly passionate about. UNICEF’s child survival strategy became the epitome of selective PHC, focused on improving survival of children through a set of targeted interventions (growth monitoring, oral rehydration therapy, breastfeeding and immunization), summarized as GOBI (Cash *et al.* 1987). Criticized as a ‘Band-Aid’, GOBI nevertheless became popular with donors, not least because it enabled them to tie their financial inputs to specific interventions and outcomes (Cueto 2004).

According to a senior Belgian maternal health specialist we interviewed, enthusiasm for GOBI’s low-cost community-based solutions engendered an ‘anti-high tech predilection’ among donors, undermining support for professionalized facility-based obstetric care, which experts saw as essential to prevent maternal deaths. It was this view that led the authors of a seminal Lancet article to ask, ‘Where is the M in MCH?’ (Rosenfield and Maine 1985), galvanizing interest in an international SMI as a counterweight to UNICEF’s Child Survival Revolution. This initiative, launched in 1987, was governed by an Inter-Agency Group with representatives from UN technical agencies (WHO, UNICEF, UNFPA), the World Bank and an NGO-based secretariat (Family Care International) and gradually expanded to incorporate other NGOs and academics (AbouZahr 2003).

The SMI was successful in bringing international attention to maternal mortality in low- and middle-income countries; within a few years, safe motherhood conferences had been held in every world region and many countries had set up dedicated maternal health programmes. However, the creation of the SMI also cemented competition between the maternal and child fields. Throughout the late 1980s and 1990s, the ‘MCH’ concept was gradually dismantled in favour of separate public health responses targeted at women and children, and competition between maternal and child health for scarce resources intensified (Lawn *et al.* 2006). Meanwhile, debates about whether resources should be invested in the provision of community-based approaches or facility-based services, and on how to balance short-term urgent demands for saving lives with long-term needs for public health system strengthening, became increasingly polarized (see McCoy *et al.* 2010).

In contrast to the selective GOBI approach, the SMI proposed a comprehensive policy agenda combining action on obstetric care, health system development and the social determinants of poor maternal health, including women’s low status (Starrs 1987). According to one of the initiative’s founders, the name ‘safe motherhood’ was strategically chosen to encompass all these issues:

One of the things that was so useful about the term ‘safe motherhood’ from the very beginning was that it’s something no one could say they were against. The most hardnosed unsentimental decision-maker or economist has a mother, sister, wife or daughter. Nobody can say they are opposed to reducing maternal mortality … and if you take the broad approach to maternal health you bring in issues not just of training of mid-wives and supplying health facilities but also the issues of education for girls, for women’s status and women’s rights in society … but you can do so in a way which is not so threatening.

Despite the SMI’s comprehensive vision, once faced with the economic crisis of the 1980s and health systems weakened by structural adjustment, many low-income country governments and donors narrowed their maternal health programmes to a limited set of community-based interventions: training of traditional birth attendants and antenatal screening for high-risk pregnancies. Ironically, these were the very interventions SMI founders had rejected as inadequate for tackling maternal mortality.

Maintaining momentum for the SMI’s broad agenda became increasingly difficult in the 1990s, especially when the SMI became implicated in the controversy that erupted at the UN International Conference on Population and Development (ICPD) in Cairo 1994 over reproductive health and rights, notably over the issue of abortion. Because the SMI had identified unsafe abortion as a main cause of maternal mortality, many conservative governments came to see safe motherhood as a ‘Trojan horse’ for the introduction of abortion rights, and withdrew their support (AbouZahr 2001). This prompted the SMI to distance itself from abortion and women’s right in favour of a more instrumental focus on preventing maternal deaths through skilled birth attendance (delivery by a professional mid-wife or doctor) and emergency obstetric care (EmOC) (Storeng & Béhague 2013).

By the end of the decade, what we term a narrative of failure had established itself within the field, with influential editorials reflecting on persistently high levels of maternal mortality and the SMI’s failure to achieve its programmatic aims and sustain political momentum (e.g. Maine and Rosenfield 1999; AbouZahr 2001). In interviews, some of those who had been active within the SMI in its early years blamed institutional weakness and a lack of leadership for lagging progress. ‘We never had a Jim Grant’, noted one such informant, referring to UNICEF’s charismatic leader in the 1980s, while another sardonically asked, ‘you ask people to cite two or three people who are shouting for maternal health in the media, in the public and they will not be able to say *one*. They won’t say anything, but for child health they will immediately say UNICEF’.

From the 1990s onwards, the SMI also struggled to respond to donors’ and policy-makers’ increasing demand for quantitative evidence of the cost-effectiveness of their investments, as we have discussed in more detail elsewhere (Béhague and Storeng 2008, Storeng and Béhague 2014). The idea of that SMI was stuck in a ‘measurement trap’ (Graham and Campbell 1992)—a negative feedback loop where measurement challenges led to lack of evidence and neglect of maternal health in research and programmes—became a dominant way of explaining the SMI’s lack of power, success and funding. The challenges posed by growing demand for evidence were reinforced by the rise of the ‘new philanthropy’, exemplified by the Gates Foundation, which instated management-style performance accountability measures to guide assessments and programming. This encouraged a bias in favour of narrow technical interventions whose value was easier to demonstrate, in place of the preceding era’s broader approach to health and social well-being (Birn 2009).

The Gates Foundation’s entry also precipitated the establishment of major global PPPs for health with which the SMI struggled to compete. The rapid rise of the Global Fund to Fight AIDS, Tuberculosis and Malaria, for instance raised concerns that ‘AIDS exceptionalism’ was fuelling neglect of maternal health, a fear at least partially substantiated by independent analyses (Crossette 2005; Shiffman 2007). While both maternal health and child survival were included among the MDGs in 2001, prominent maternal health experts recalled how the Gates Foundation’s support for child health through the establishment of the vaccine alliance GAVI in 2000 and Saving Newborn Lives (later the Healthy Newborn Partnership) in 2004 marginalized maternal health.

Around the same time, international debate about the need to increase ‘aid-effectiveness’ intensified due to concerns that heavy emphasis on project-based and donor-driven funding was distorting national planning and priority-setting. While some donors had supported new aid modalities like sector wide approaches and direct budget support to low-income country governments in the 1990s, major donors including USAID, rejected them, and as a result international initiatives for safe motherhood and child survival had continued to operate in parallel, with separate budgets and management structures (Standing 2002). The proliferation of disease-specific PPPs reinforced vertical approaches and fitted poorly with longer-term planning and financing of health sectors. This fed into more general disquiet about the poor effectiveness of international development assistance, giving rise to ‘ownership’, ‘alignment’, ‘harmonization’, ‘managing results’ and ‘mutual accountability’ as core ideals that should underpin international aid practices (Mosse and Lewis 2005). These principles, contained within the Paris Declaration on Aid Effectiveness, were endorsed by more than 100 signatories from donor and low-income country governments, multilateral donor agencies, regional development banks and international organizations (OECD DAC 2005). All the agencies involved in the SMI supported the Paris Declaration, not least because disease-specific donor-driven projects with separate budgets often translated into neglect of cross-cutting health system issues deemed necessary for reducing maternal mortality (Task Force on Child Health and Maternal Health 2005). At the same time, it became clear at policy and research events we attended around this time that the Declaration also signalled that the SMI’s funding was under threat, as donors became explicit about wanting to rationalize and fund fewer global health initiatives.

### Moving towards partnership

Together, these somewhat contradictory tendencies—greater competition to survive as a single initiative combined with pressure to harmonize and integrate development efforts—converged to create an impetus for the SMI to find strategies to protect the status that maternal health had achieved through being assigned as one of the MDGs. Partnership formation was part of such a strategy.

A preamble to the establishment of the PMNCH was the creation of the Partnership for Maternal and Newborn Health in 2004. Already dismayed that MDG 4 on child survival was attracting greater commitment than MDG 5 on maternal health, SMI leaders we interviewed explained that they feared that the promotion of newborn survival as a subsidiary goal of child survival would further skew prioritization. They therefore pre-emptively proposed a merger with the newly formed Healthy Newborn Partnership, successfully persuading its leaders that a partnership would be mutually beneficial, since newborn survival is so closely related with safe delivery and women’s survival that any efforts to improve maternal health would also benefit newborn survival.

With newborn survival as the link between MDGs 4 and 5, a consensus soon emerged within the UN agencies and the broader advocacy communities that it would ‘make sense’ to incorporate the existing child survival community into the new partnership. Meanwhile, there were ongoing talks about forming a new Child Survival Partnership to revive the Child Survival Revolution of the 1980s and drive progress on MDG 4. However, both maternal and child health specialists we interviewed recalled that certain of those donors who were particularly strongly committed to the Paris Declaration, such as the UK Department for International Development (DFID), made it clear that they would refuse to handle requests from multiple partnerships working on what were arguably inter-linked goals. According to a SMI member who later became part of the PMNCH’s steering committee, donors ‘really wanted to see this field co-ordinated and connected and they didn’t want several sets of transaction costs for the funding that they ‘did’ want to put into the work’. As another SMI leader recalled, ‘they said, “either you merge or die, you are not funded,” so we decided to merge’.

The PMNCH was officially established at the UN General Assembly in 2005. Its vision, institutional structure and strategic objectives had been debated at the high-level meeting ‘Lives in the Balance’ in New Delhi, India in April 2005, which we attended, coinciding with the launch of the WHO’s World Health Report: Make every mother and child count (WHO 2005). The Delhi Declaration drafted at the meeting called on all countries to ‘orient their national and sub-national development plans and budgets to fully achieve the maternal and child health MDGs by 2015’ (PMNCH 2005).

The PMNCH was hosted by the WHO in Geneva. By the end of 2005, a Board and advisory committees had been assembled, drawing representatives from across multiple constituencies: donors and foundations, health care professionals, multi-lateral agencies, NGOs, partner countries, researchers and academics, and the private sector. The inclusion of donors and private-sector actors within the governance structure was a major shift for the SMI, which had excluded donors in a bid to secure its independence to work on controversial issues like abortion. Like many PPPs, the PMNCH aimed to promote knowledge and innovation to advance policy, service delivery and financing, do advocacy and consolidate resources and promotion accountability. But it differed in its explicit endorsement of the Paris Declaration on aid-effectiveness; the PMNCH promised more appropriate financial and technical assistance to low-income countries, including greater country ‘ownership’ of national policy processes and global advocacy and policy advice aligned with national health and development plans (PMNCH 2009a). The PMNCH intended to provide ‘catalytic’ financial and technical support to countries to implement a ‘continuum of care’ to accelerate progress towards MDGs 4 and 5. The new partnership received funding from the governments of Australia, Canada, Germany, Italy, Netherlands, Norway, Sweden, UK and the USA, the World Bank, UNICEF and the Gates and MacArthur Foundations, as well as ‘in-kind’ support from many other partners (PMNCH 2015).

### The promise of partnership

The PMNCH officially incorporated SMI actors and expertise and thus brought an end to the SMI as it had existed, yet many safe motherhood advocates accepted this development as inevitable and even welcome. ‘It needs to happen’, explained a senior maternal health specialist in an interview on the eve of the PMNCH’s launch, ‘because as a single topic we can’t fight this alone, we can’t survive’. Many hoped that the PMNCH could help strengthen global priority for maternal health through increased funding and stronger institutional leadership; they hoped that Francisco Songane, the PMNCH’s first director and an obstetrician and former Minister of Health from Mozambique, would become the global maternal health champion the SMI had lacked. The PMNCH would also strengthen global advocacy, several anticipated. The Partnership claimed that ‘more than 6 million maternal, newborn and child deaths would be averted yearly if essential maternal, newborn and child health and nutrition interventions are implemented at scale’ (PMNCH 2008). This was, according to one American communication specialist, simply a ‘much better advocacy argument’ than the SMI’s call for action to save 500 000 lives (the annual global maternal death estimate at the time). As a UK-based maternal health epidemiologist put it, ‘It makes sense to bung in the babies for the numbers game’ (Storeng and Béhague 2014).

Both maternal and child health specialists were optimistic that the PMNCH would develop and generate support for a coherent set of policy recommendations, overcoming what one editorial labelled the ‘false and damaging dichotom[ies]’ between facility-based and community-based approaches that had existed for maternal and child health, respectively, since the 1980s (Lawn *et al.* 2006, p. 1474). As such, they were enthusiastic about the ‘continuum of care’ framework the PMNCH intended to help countries implement. The continuum was an integrated ‘life cycle’ approach to health improvement linking, in time, care from pregnancy through birth, newborn and young child health and, in place, the various levels of home, community and health facilities (PMNCH 2009b). This framework had important limitations; it focused heavily on clinical interventions and ignored women’s health outside of pregnancy and the social determinants of health (cf. Yamin and Boulanger 2014). Yet, maternal health advocates welcomed its alignment of disease-specific approaches and attention to cross-cutting issues like infrastructure and human resources in the health sector, as well as its support for EmOC and skilled birth attendance, issues they had long struggled to generate support for through the SMI.

### The challenges of institutional and policy integration

Despite the many clear strategic and ideological benefits of integration, the complexities of advancing multipronged initiatives quickly came to the fore. For example, although the PMNCH aimed to be inclusive, some members felt that they were not being well represented. One maternal health NGO representative complained that the ‘elitist’ nature of the Inter-Agency Group—which had been dominated by small and select group of UN agency senior advisors—was being reproduced:

From what I understand, all the meetings that have been held so far—the high-level meetings—are kind of elitist. Even though they want to have these working groups, I don’t think that there’s an attempt by the Partnership to open up the groups and invite people in from different organizations that were not part of this elitist group before.

Two years into the PMNCH, a senior Belgian maternal health epidemiologist similarly lamented, ‘I have not talked to anybody that becomes shiny eyed when they talk about the global Partnership. [No one seems to feel that this is] something new, something big, something strong where we really get together’.

Coordination problems quickly tarnished the PMNCH’s external credibility. At an evidence session for the UK House of Commons International Development Committee inquiry into maternal health we attended in November 2007, committee members expressed confusion about its remit, questioning why the WHO was simultaneously host to the PMNCH and to two separate departments—Making Pregnancy Safer and Reproductive Health and Research—working on women’s health. The committee’s report concluded: ‘it is far from clear to us how the UN divides up responsibility for different aspects of maternal, newborn and child health…the overlapping remits between agencies has contributed to a lack of confidence in the UN as a global leader’ (House of Commons Select Committee on International Development, 2008, p. 25). Meanwhile, a senior WHO health system specialist we interviewed was critical of institutional wrangling in establishing the PMNCH: ‘strategy is [being] sacrificed for structural considerations, which relates to “who’s going to host the secretariat” and junk like that, which doesn’t really do a whole lot for mothers anywhere’.

Maternal health advocates, in turn, worried primarily about their own weak position within the PMNCH. A senior UK-based maternal health researcher, for instance, reflected: ‘When brought together with the child health group, [we] have always been poor relatives…always the less substantiated and less well supported group…and the same is true in the Partnership’. Former SMI representatives similarly alleged that despite the global rhetoric of ‘integration’, PMNCH donors continued to favour child health. ‘We are the last of the trio to get money from Gates’, said a senior UNFPA maternal health advisor, explaining that the Foundation had channelled $25 million through the PMNCH for child health programmes in Africa even though the Partnership had publicly committed to aligned funding for maternal and child health. Similarly, a ‘Global Business Plan’ devised by the Norwegian Prime Minister and administered through the PMNCH had focused narrowly on child survival until safe motherhood advocates eventually persuaded the Board to include maternal health (see PMNCH 2009c).

Some informants attributed such apparent child health bias to certain donors' reticence to be associated with more politically sensitive aspects of women’s health, especially abortion. The UNFPA advisor cited above explained that his organization had already lost USAID funding because of its work on abortion, and said he feared that the Gates Foundation’s involvement in the PMNCH would exacerbate the situation: ‘We haven’t received a single dollar [from the US] since Bush… so, maternal health is linked to women’s health and to abortion and some donors are sensitive [to this], even… Gates…Melinda Gates is very religious, you know’. Others worried that the newborn health lobby within the PMNCH was legitimating such attitudes by, as one senior UK-based NGO advisor put it, ‘consciously or unconsciously using some of the same language that anti-abortionists use’. Although there has historically been deep ambivalence within the SMI about whether or not to remain neutral in pro-choice debates (Storeng and Béhague 2013), a number of maternal health advocates who were ideologically committed to reproductive rights began to question the strategic value of the alliance with the newborn health lobby. ‘I’m inclined to think that people have gone for this new approach because newborns are something that everyone coos over and it may be a way to kick-start new political will and get more money into the field’, said the NGO advisor cited above, ‘but I think there are risks that one ought to be aware of’.

The maternal health coalition’s status as the ‘poor relative’ within the PMNCH was also seen as a reflection of continued lack of commitment towards the integrated public health approaches needed to address maternal mortality. A senior researcher claimed that key PMNCH members pay lip service to maternal health, but still hold ‘prejudice against the complexity of maternal health’ and question the cost-effectiveness of key maternal health interventions like skilled birth attendance:

You know, I’ve seen emails that probably shouldn’t come to me that talk about the ‘elephant in the room’, which, you know, in some ways summarizes some people’s perspectives of maternal [health]. There is this attitude that ‘we don’t have the evidence for maternal interventions but we just have to do it’. I’m not going to name names, but there are several key members—donors—within the Partnership who have, implicitly, if not explicitly, stated this.

Others implied that child and newborn health advocates within the Partnership were complicit in creating this situation by distorting the continuum of care concept to promote their own preference for community-based care and selective ‘magic bullets’. For example, a senior American maternal health epidemiologist accused newborn health advocates of being insensitive to the potentially detrimental policy impact of pushing for interim community-based solutions:

I think of myself as being pretty balanced on this issue…but I find myself reacting to the newborn people. I have heard [one advocate], who is like Mr. Neonate, say, ‘you know, blah, blah, blah, skilled attendance in countries like Bangladesh where there aren’t enough providers and this, that and the other, there are things that we could be doing right now *in the home* to save newborns’. This expression sets me off: ‘yes, we need skilled attendance, but in the *meantime* …’ because what ‘in the meantime’ means to a policy-maker is ‘do nothing’.

Another senior maternal health epidemiologist similarly described how her suggestion during a committee meeting to foreground skilled birth attendance within the continuum of care framework was immediately rejected: ‘I received quite negative feedback from the neonatal and child people, saying that, ‘yes of course you need a skilled attendant but it’s a long-term initiative. We need short-term intermediate solutions’. This came up repeatedly. And I’m uneasy with that claim’.

### Self-protective responses and internal fragmentation

Several years into the new partnership, the on-going force of the tensions that had divided the child and maternal health communities since the 1980s put safe motherhood advocates in a bind. On the one hand, they were committed—institutionally, financially and conceptually—to the process of partnership-building that they had initiated. On the other, many expressed disappointment that their expectations had not been met, and some had started to fear that the PMNCH could even be harmful to maternal health interests and emphasized the importance of ‘protecting’ maternal health’s position. ‘I would say that we have to fight, to constantly remind people that the Partnership is for MDG 4 and 5’, said UNFPA’s senior maternal health advisor.

A variety of self-protective practices emerged in response to such perceptions. For example, some sought to challenge what they saw as the distortion of the continuum of care concept. As a senior UK-based maternal health researcher explained:

I think we need to make sure, all of us collectively, we need to make sure that the M in MNCH does not get lost. We have to be sure that, because maternal mortality is a longer-term intervention and we have to look at issues of human resources and at strengthening health infrastructure, that donors and governments don’t just go for the quick-wins that are easier to do and have a quick impact, but [rather that they] have a commitment to the longer-term interventions.

Others, by contrast, responded more directly to the strategic demands of generating funds and political attention by advancing a series of maternal health-specific campaigns, even though this was in tension with the PMNCH’s aim of an integrated policy approach. A key example was the Women Deliver campaign spearheaded by activists from within UNFPA and the NGO Family Care International (which had served as the SMI’s secretariat). This campaign culminated in a 3000-delegate conference in London in 2007 which we attended, calling for donor prioritization of *maternal* health through the slogan ‘Invest in women—it pays’. While such issue-specific advocacy is not necessarily contrary to the strategic objectives of the PMNCH, it is telling that the conference stayed clear of the ‘continuum of care’ rhetoric and instead promoted key interventions to reduce maternal mortality, and framed investments in *maternal* health as the fulcrum of achieving *all* the MDGs, including child survival and poverty reduction. The conference organizers adopted an instrumental, neoliberal economic logic that appealed to donors, but that was in tension with the Partnership’s integration rhetoric and jarred with the feminist, social justice discourse the SMI had originally promoted.

## Discussion: a global health success?

To what extent has the PMNCH advanced integration of policy approaches at the global level? The Partnership has certainly covered considerably ground. As we have shown elsewhere, key players in maternal and child health have spearheaded wide-ranging research agendas that are resolutely committed to integration (Béhague and Storeng 2013), and they are doing this within the parameters set by the Partnership (see, for example, Stenberg *et al*. 2014). Yet, our case study shows that the rhetoric of integration often masks continued structural drivers (McCoy et al. 2013) that bias the global health field towards vertical goals.

The ability to generate funding is often taken as evidence of the success of global health PPPs (Shiffman *et al.* 2015). Indeed, in outlining its achievements, the PMNCH emphasizes that its annual funded budgets rose from around US$6 million in 2009 to more than US$10 million in 2015, and that it has leveraged additional resources through global initiatives it has supported (PMNCH 2015). For example, the Global Strategy for Women’s and Children’s Health launched in 2010 is said to have generated an estimated US$20 billion in new and additional money to women’s and children’s health (PMNCH 2014). Independent analyses confirm that funding to MNCH increased substantially in the first few years of the new partnership, but this increase was proportional to increases in funding for health in general and cannot necessarily be attributed to the PMNCH’s efforts (Pitt *et al.* 2010). Moreover, there are serious concerns about sustainability (Hsu *et al.* 2012; Horton 2014).

Crucially, the extent to which global funding has translated into integrated policy approaches at global or national levels is difficult to establish. The increased funding to newborn health, for instance, has focused on research, rather than large-scale implementation (Pitt *et al.* 2012). While wider programmes targeting maternal and child health, or even general primary health care programmes, mention ‘newborns’ more often in their descriptions, there is no evidence that this has led to concrete integrated programing and joined-up thinking, or the health system strengthening that is needed to sustain integration (Pitt, personal communication 25.04.14). In fact, most public health interventions remain targeted at women, newborns or children, and few evaluations of interventions consider the impact on more than one of these groups (McCoy *et al.* 2010).

The PMNCH strongly implies that health improvements can be attributed to its efforts, saying that since it was formed in 2005, ‘global attention and governments have turned to support and improve the health of women’s and children’s health globally, and change and improve [maternal and child mortality] statistics’ (PMNCH 2014). However, it is very difficult to attribute health improvement to specific global-level initiatives; the attribution of ‘lives saved’ to specific PPPs is not only prone to overestimation, but might also negatively affect the overall governance of health systems and reinforce vertical programmes (McCoy *et al.* 2013).

At its core, a key challenge for institutional integration lies in the notion of ‘partnership’ to which few ‘partners’ seem to subscribe and many describe as ‘elitist’. It is problematic that PMNCH members still primarily identify with one area of expertise, referring to themselves and their colleagues as either maternal health, newborn health or child survival ‘people’. The fact that political scientists continue to analyse ‘issue attention’ to newborn and maternal survival separately rather than as part of broader political struggles (e.g. Shiffman 2010; Tinker *et al.* 2010; Smith *et al.* 2014) also suggests that disease-based approaches are not being challenged in any significant way. Since the PMNCH was launched, the leading medical journal *The Lancet* has even published separate series focused on maternal, newborn and child health, without corresponding attention to integrated policy approaches. Significantly, the PMNCH’s policy discourse remains focused on technical aspects of health improvement and an interpretation of integration focused on the parallel scaling up of disease-specific interventions, eschewing national governments' role in public health or the social determinants of health and gender issues that were so central in the SMI’s initial integrated policy agenda. After a decade of defining maternal health primarily in terms of maternal mortality, the Partnership claims that it now ‘belatedly’ focuses on reproductive health and has even started to define its focus as ‘RMNCH’ to incorporate the ‘R’ of ‘reproductive health’ within the MNCH concept (Bustreo *et al.* 2012). Still, its public profiling makes little if any reference to its position on and work within core reproductive health issues like unwanted pregnancy, unsafe abortion or gender-based violence and discrimination, and it is not clear to what extent self-proclaimed reproductive health advocates consider themselves to be part of the Partnership.

## Conclusion

Our case study contributes to the emerging ethnographic literature on the rhetoric and reality of PPPs by showing the complexity of developing and maintaining partnership between actors with different histories, social configurations and approaches to health improvement. While key PMNCH actors claim there is now consensus about the value of partnership, we have shown that its history has also been characterized by competitive tensions, which elicited protective responses, not least from the safe motherhood contingent. The speed and intensity of these responses were no doubt informed by the SMI’s enduring struggle to assert itself, including its experience since the 1980s of seeing maternal health subordinated to child health (Storeng 2010). Ironically, however, such self-protective practices may accentuate the kind of fragmentation to global health governance, financing and policy solutions that the Partnership was intended to challenge.

Although the PMNCH has laudably embraced the aims of integration of policy approaches and institutional governance, global health partnerships remain under pressure to appeal to donors by being issue-specific and quick-results oriented (Buse and Harmer 2007). As our findings underscore, the cost of operating within an extremely competitive global health arena may be at least a partial renouncement of ambitions to broader social transformations in favour of narrower, but feasible and ‘sellable’ interventions (Irwin and Scali 2007, p. 243). Until the PMNCH—and other PPPs—take a more radical approach to the core structures of global health governance within which they must operate, integration is likely to remain a difficult-to-implement ideal.

## References

[czw023-B1] AbouZahr C. 2001 Cautious champions: international agency efforts to get safe motherhood onto the agenda. Studies in Health Services Organisation and Policy 17: 387–414.

[czw023-B2] AbouZahr C. 2003 Safe motherhood: a brief history of the global movement 1947-2002. British Medical Bulletin 67: 13–25. 14711751 10.1093/bmb/ldg014

[czw023-B3] Atun R de Jongh T Secci F Ohiri K Adeyi O. 2010 Integration of targeted health interventions into health systems: a conceptual framework for analysis. Health Policy and Planning 25: 104–11. 19917651 10.1093/heapol/czp055

[czw023-B64] Béhague DP Storeng KT. 2008 Collapsing the vertical-horizontal divide: an ethnographic study of evidence-based policymaking in maternal health. American Journal of Public Health 98(4): 644–9. 18309123 10.2105/AJPH.2007.123117 PMC2376990

[czw023-B4] Béhague D Tawiah C Rosato M Some T Morrison J. 2009 Evidence-based policy-making: the implications of globally-applicable research for context-specific problem-solving in developing countries. Social Science & Medicine 69(10): 1539–46. 19781839 10.1016/j.socscimed.2009.08.006

[czw023-B250] Béhague DP Storeng KT. 2013 Pragmatic politics and epistemological diversity: the contested and authoritative uses of historical evidence in the Safe Motherhood Initiative. Evidence & Policy: A Journal of Research, Debate and Practice 9: 65–85.

[czw023-B5] Birn AE. 2009 The stages of international (global) health: histories of success or successes of history? Global Public Health 4: 50–68. 19153930 10.1080/17441690802017797

[czw023-B6] Birn AE. 2014 Philanthrocapitalism, past and present: the Rockefeller Foundation, the Gates Foundation, and the setting(s) of the international/global health agenda. Hypothesis 12: e8.

[czw023-B7] Brown TM Cueto M Fee E. 2006 The World Health Organization and the transition from “international” to “global” public health. American Journal of Public Health 96: 62–72. 16322464 10.2105/AJPH.2004.050831 PMC1470434

[czw023-B8] Buffardi AL. 2014 From polarisation to practice: puzzles and insights on integrated approaches from public health professionals. Global Public Health 9: 741–51. 24992263 10.1080/17441692.2014.929724

[czw023-B9] Buse K Harmer AM. 2007 Seven habits of highly effective global public-private health partnerships: practice and potential. Social Science & Medicine 64: 259–71. 17055633 10.1016/j.socscimed.2006.09.001

[czw023-B10] Bustreo F Requejo JH Merialdi M Presern C Songane F. 2012 From safe motherhood, newborn, and child survival partnerships to the continuum of care and accountability: moving fast forward to 2015. International Journal of Gynaecology and Obstetrics 119(Suppl 1): S6–8. 22883915 10.1016/j.ijgo.2012.03.008

[czw023-B11] Cash R Keusch G Lamstein J (eds). 1987 Child Health & Survival: The UNICEF GOBI-FFF Program. London: Croom Helm.

[czw023-B12] Church K Wringe A Lewin S 2015 Exploring the feasibility of service integration in a low-income setting: a mixed methods investigation into different models of reproductive health and HIV care in Swaziland. PLoS One 10: e0126144. 25978632 10.1371/journal.pone.0126144 PMC4433110

[czw023-B13] Crossette B. 2005 Reproductive health and the Millennium Development Goals: the missing link. Studies in Family Planning 36: 71–9. 15828526 10.1111/j.1728-4465.2005.00042.x

[czw023-B14] Cueto M. 2004 The origins of primary health care and selective primary health care. American Journal of Public Health 94: 1864–74. 15514221 10.2105/ajph.94.11.1864 PMC1448553

[czw023-B15] Doyle C Patel P. 2008 Civil society organisations and global health initiatives: problems of legitimacy. Social Science & Medicine 66: 1928–38. 18291566 10.1016/j.socscimed.2007.12.029

[czw023-B16] Eckl J. 2014 The power of private foundations: Rockefeller and Gates in the struggle against malaria. Global Social Policy 14: 91–116.

[czw023-B17] Graham WJ Campbell OM. 1992 Maternal health and the measurement trap. Social Science & Medicine 35: 967–77. 1411704 10.1016/0277-9536(92)90236-j

[czw023-B18] Grebe E. 2015 The ambiguities of the 'partnership' between civil society and the state in Uganda's AIDS response during the 1990s and 2000s as demonstrated in the development of TASO. Global Public Health 1–17. 10.1080/17441692.2015.1062121 26251294

[czw023-B19] Harmer A. 2011 Understanding change in global health policy: ideas, discourse and networks. Global Public Health 6: 703–18. 20924870 10.1080/17441692.2010.515236

[czw023-B20] Harmer A Spicer N Aleshkina J 2012 Has Global Fund support for civil society advocacy in the Former Soviet Union established meaningful engagement or 'a lot of jabber about nothing'? Health Policy & Planning 28(3): 299–308. 22767433 10.1093/heapol/czs060

[czw023-B21] Horton R. 2014 Offline: Ban Ki-moon's global health initiative in jeopardy. The Lancet 383: 292.

[czw023-B22] House of Commons Select Committee on International Development. 2008 *Maternal health. Fifth report of session 2007-2008* London, The Stationary Office by order of the House of Commons. http://www.parliament.the-stationery-office.co.uk/pa/cm200708/cmselect/cmintdev/66/6602.htm, accessed March 16, 2016.

[czw023-B23] Hsu J Pitt C Greco G Berman P Mills A. 2012 Countdown to 2015: changes in official development assistance to maternal, newborn, and child health in 2009/10, and assessment of progress since 2003. The Lancet 380: 1157–68. 10.1016/S0140-6736(12)61415-9 23000291

[czw023-B24] Irwin A Scali E. 2007 Action on the social determinants of health: a historical perspective. Global Public Health 2: 235–56. 19283626 10.1080/17441690601106304

[czw023-B25] Kapilashrami A McPake B. 2012 Transforming governance or reinforcing hierarchies and competition: examining the public and hidden transcripts of the Global Fund and HIV in India. Health Policy & Planning 28(6):626–35. 23144229 10.1093/heapol/czs102

[czw023-B26] Kapilashrami A O'Brien O. 2012 The Global Fund and the re-configuration and re-emergence of 'civil society': widening or closing the democratic deficit? Global Public Health 7: 437–51. 22239445 10.1080/17441692.2011.649043

[czw023-B27] Lawn JE Tinker A Munjanja SP Cousens S. 2006 Where is maternal and child health now? The Lancet 368: 1474–7. 10.1016/S0140-6736(06)69387-2 17071267

[czw023-B28] Magnussen L Ehiri J Jolly P. 2004 Comprehensive versus selective primary health care: lessons for global health policy. Health Affairs 23: 167–76. 15160814 10.1377/hlthaff.23.3.167

[czw023-B29] Maine D Rosenfield A. 1999 The safe motherhood initiative: why has it stalled? American Journal of Public Health 89: 480–2. 10191784 10.2105/ajph.89.4.480 PMC1508874

[czw023-B30] McCoy D. 2009 Global health initiatives and country health systems. The Lancet 374: 1237. 10.1016/S0140-6736(09)61778-5 19819383

[czw023-B31] McCoy D Jensen N Kranzer K Ferrand RA Korenromp EL. 2013 Methodological and policy limitations of quantifying the saving of lives: a case study of the Global Fund's approach. PLoS Medicine 10: e1001522. 24130461 10.1371/journal.pmed.1001522 PMC3794859

[czw023-B32] McCoy D McGoey L. 2011 Global health and the gates foundation—in perspective In: Rushton S Williams OW (eds). Partnerships and Foundations in Global Health Governance. Basingstoke: Palgrave.

[czw023-B33] McCoy D Storeng KT Filippi V 2010 Maternal, neonatal and child health interventions and services: moving from knowledge of what works to systems that deliver. International Health 2: 97–8. 10.1016/j.inhe.2010.03.005 24037704

[czw023-B34] Mosse D Lewis D (eds). 2005 The Aid Effect: Giving and Governing in International Development. London: Pluto Press.

[czw023-B35] OECD DAC. 2005 *Paris Declaration on Aid Effectiveness: Ownership, Harmonisation, Alignment, Results and Mutual Accountability*. Paris: OECD High-Level Forum.

[czw023-B36] Pitt C Greco G Powell-Jackson T Mills A. 2010 Countdown to 2015: assessment of official development assistance to maternal, newborn, and child health, 2003–08. The Lancet 376: 1485–96. 10.1016/S0140-6736(10)61302-5 20850869

[czw023-B37] Pitt C Lawn JE Ranganathan M Mills A Hanson K. 2012 Donor funding for newborn survival: an analysis of donor-reported data, 2002-2010. PLoS Medicine 9: e1001332. 23118619 10.1371/journal.pmed.1001332 PMC3484125

[czw023-B58] PMNCH. 2005 Delhi Declaration on Maternal, Newborn and Child health. Geneva, The Partnership for Maternal, Newborn and Child Health www.who.int/world-health-day/previous/2005/delhi_declaration.pdf, accessed March 14, 2016.

[czw023-B38] PMNCH. 2008 *A Global Call for G8 Leaders and Other Donors to Champion Maternal, Newborn and Child Health*. Geneva: Partnership for Maternal, Newborn and Child Health.

[czw023-B39] PMNCH. 2009a *Partnership Vision: Invest, Deliver, Advance* [Online]. http://www.who.int/pmnch/en/, accessed 16 March 2009.

[czw023-B40] PMNCH 2009b *Strategy and Workplan 2009-2011*. Geneva: The Partnership for Maternal, Newborn and Child Health.

[czw023-B41] PMNCH. 2009c *The Global Business Plan for Millennium Development Goals 4 and 5* [Online]. http://www.who.int/pmnch/activities/globalbusinessplan/en/index.html, accessed 19 February 2014.

[czw023-B42] PMNCH. 2014 *PMNCH History* [Online]. http://www.who.int/pmnch/about/history/en/, accessed 19 February 2014.

[czw023-B43] PMNCH. 2015 *Funders* [Online]. http://www.who.int/pmnch/about/funders/en/, accessed 25 September 2015.

[czw023-B44] Ravishankar N Gubbins P Cooley RJ 2009 Financing of global health: tracking development assistance for health from 1990 to 2007. The Lancet 373: 2113–24. 10.1016/S0140-6736(09)60881-3 19541038

[czw023-B45] Reich MR (ed). 2002 Public-Private Partnerships for Public Health*.* Cambridge, MA: Harvard Center for Population and Development Studies (Distributed by Harvard University Press).

[czw023-B46] Rosenfield A Maine D. 1985 Maternal mortality—a neglected tragedy. Where is the M in MCH? The Lancet 2: 83–5. 10.1016/s0140-6736(85)90188-6 2861534

[czw023-B47] Sabatier PA Jenkins-Smith HC. 1993 Policy Change and Learning: An Advocacy Coalition Approach. Boulder, CO: Westview Press.

[czw023-B48] Shiffman J. 2007 Has donor prioritization of HIV/AIDS displaced aid for other health issues? Health Policy & Planning 23: 95–100. 18156161 10.1093/heapol/czm045

[czw023-B49] Shiffman J. 2010 Issue attention in global health: the case of newborn survival. The Lancet 375: 2045–9. 10.1016/S0140-6736(10)60710-6 20569844

[czw023-B50] Shiffman J Quissell K Schmitz HP 2015 A framework on the emergence and effectiveness of global health networks. Health Policy & Planning Epub ahead of print: 1–14. 10.1093/heapol/czu046 PMC4954553 26318679

[czw023-B51] Smith SL Shiffman J Kazembe A. 2014 Generating political priority for newborn survival in three low-income countries. Global Public Health 9: 538–54. 24766101 10.1080/17441692.2014.904918

[czw023-B52] Standing H. 2002 An overview of changing agendas in health sector reforms. Reproductive Health Matters 10: 19–28. 12557639 10.1016/s0968-8080(02)00089-7

[czw023-B53] Starrs A. 1987 *Preventing the Tragedy of Maternal Deaths: Report of the Safe Motherhood Conference*, Nairobi, Kenya, February 1987. New York: Family Care International.

[czw023-B100] Stenberg K Axelson H Sheehan P 2014 Advancing social and economic development by investing in women's and children's health: a new Global Investment Framework. The Lancet, 383, 1333–54. 10.1016/S0140-6736(13)62231-X 24263249

[czw023-B54] Stone D. 2002 Introduction: global knowledge and advocacy networks. Global Networks 2: 1–12.

[czw023-B65] Storeng KT. 2010 Safe motherhood: the making of a global health initiative (PhD Dissertation). PhD, University of London.

[czw023-B66] Storeng KT Béhague DP 2013 Evidence-based advocacy and the reconfiguration of rights language in safe motherhood discourse. Health rights in global context: Geneaologies and Anthropologies. A. Mold and D. Reubi. Routledge, London and New York: 149–168.

[czw023-B55] Storeng KT. 2014 The GAVI Alliance and the ‘Gates approach’ to health system strengthening. Global Public Health 9(8): 865–879. 25156323 10.1080/17441692.2014.940362 PMC4166931

[czw023-B67] Storeng KT Béhague DP. 2014 “Playing the Numbers Game”: Evidence-based Advocacy and the Technocratic Narrowing of the Safe Motherhood Initiative. Medical Anthropology Quarterly 28(2): 260–279. 24599672 10.1111/maq.12072 PMC4314706

[czw023-B56] Task Force on Child Health and Maternal Health. 2005 *Who's Got the Power? Transforming Health Systems for Women and Children*. London: UN Millennium Project.

[czw023-B57] Taylor EM Harper I. 2014 The politics and anti-politics of the global fund experiment: understanding partnership and bureaucratic expansion in Uganda. Medical Anthropology 33: 206–22. 24761975 10.1080/01459740.2013.796941

[czw023-B59] Tinker A Parker R Lord D Grear K. 2010 Advancing newborn health: the saving newborn lives initiative. Global Public Health 5: 28–47. 19851911 10.1080/17441690903286572 PMC2790805

[czw023-B60] Travis P Bennett S Haines A 2004 Overcoming health-systems constraints to achieve the Millennium Development Goals. The Lancet 364: 900–6. 10.1016/S0140-6736(04)16987-0 15351199

[czw023-B61] WHO. 2005 *World Health Report 2005. Make Every Mother and Child Count*. Geneva: World Health Organization

[czw023-B62] WHO and UNICEF. 1978 *Declaration of Alma-Ata. International Conference on Primary Health Care*, Alma-Ata, USSR, 6–12 September 1978. Geneva. World Health Organization.

[czw023-B63] Yamin AE Boulanger VM. 2014 Why global goals and indicators matter: the experience of sexual and reproductive health and rights in the Millennium Development Goals. Journal of Human Development and Capabilities 15(2-3): 218–231.

